# Detection of astrocytic slow oscillatory activity and response to seizurogenic compounds using planar microelectrode array

**DOI:** 10.3389/fnins.2022.1050150

**Published:** 2023-01-10

**Authors:** Taeko Kuroda, Naoki Matsuda, Yuto Ishibashi, Ikuro Suzuki

**Affiliations:** Department of Electronics, Graduate School of Engineering, Tohoku Institute of Technology, Sendai, Japan

**Keywords:** MEA - microelectrode array, astrocyte, seizure, human, culture, toxicology, iPSC (induced pluripotent stem cell), slow-oscilatory activity

## Abstract

Since the development of the planar microelectrode array (MEA), it has become popular to evaluate compounds based on the electrical activity of rodent and human induced pluripotent stem cell (iPSC)-derived neurons. However, there are no reports recording spontaneous human astrocyte activity from astrocyte-only culture sample by MEA. It is becoming clear that astrocytes play an important role in various neurological diseases, and astrocytes are expected to be excellent candidates for targeted therapeutics for the treatment of neurological diseases. Therefore, measuring astrocyte activity is very important for drug development for astrocytes. Recently, astrocyte activity has been found to be reflected in the low-frequency band < 1 Hz, which is much lower than the frequency band for recording neural activity. Here, we separated the signals obtained from human primary astrocytes cultured on MEA into seven frequency bands and successfully recorded the extracellular electrical activity of human astrocytes. The slow waveforms of spontaneous astrocyte activity were observed most clearly in direct current potentials < 1 Hz. We established nine parameters to assess astrocyte activity and evaluated five seizurogenic drug responses in human primary astrocytes and human iPSC-derived astrocytes. Astrocytes demonstrated the most significant dose-dependent changes in pilocarpine. Furthermore, in a principal component analysis using those parameter sets, the drug responses to each seizurogenic compound were separated. In this paper, we report the spontaneous electrical activity measurement of astrocytes alone using MEA for the first time and propose that the MEA measurement focusing on the low-frequency band could be useful as one of the methods to assess drug response *in vitro*.

## Significance statement

Planar microelectrode array (MEA) is recognized as a useful method for evaluating the toxicity of compounds to *in vitro* human neurons. However, there are no reports directly recording spontaneous human astrocyte activity from astrocyte-only culture sample by MEA, and no drug evaluation methods are established. Here, we successfully recorded extracellular electrical activity of human astrocytes using MEA in low-frequency band. Furthermore, we established a method to assess the activity of primary human astrocytes and human iPSC-derived astrocytes, and evaluated astrocyte drug responses to five seizurogenic compounds. We expect that the MEA-based system for assessing astrocyte activity will not only provide new insights into the mechanisms of neurological diseases, but will also help in the evaluation of drug efficacy in the drug development for diseased astrocytes.

## Introduction

The microelectrode array (MEA) is a planar substrate embedded with an array of microelectrodes capable of measuring extracellular potentials. In 1972, Thomas et al. reported activity recordings of cultured cells using MEA (Thomas et al., 1972). Since then, MEA has been used to record from a wide variety of neuronal preparations (Gross et al., 1977; Gross, 1979; Pine, 1980). Furthermore, improvements to the MEA have made it possible to measure from brain slices as well as from cultured neurons (Jobling et al., 1981; Wheeler and Novak, 1986; Novak and Wheeler, 1988). Since then, active research has been conducted mainly using rodent neurons and brain slices. With the development of human induced pluripotent stem cells (iPSCs), it is possible to induce human neurons from iPSCs, and human neural function can be evaluated *in vitro* (Amin et al., 2016; Odawara et al., 2016, 2022; Frega et al., 2017; Grainger et al., 2018; Autar et al., 2022; Pré et al., 2022).

Microelectrode array measurement is a noninvasive, high-temporal-resolution method and can simultaneously measure multiple points of neural network activity. MEA has been proposed as a high-throughput, accurate, and rapid screening method for toxicity testing. Indeed, studies using rodent neurons and human iPSC-derived neurons have reported the usefulness of the MEA method as an evaluation system for identifying seizurogenic compounds (Johnstone et al., 2010; Bradley et al., 2018; Odawara et al., 2018; Tukker et al., 2018, 2020; Fan et al., 2019). We reported that co-culturing neurons and astrocytes on MEA promotes early maturation of neural networks and enhances drug responsiveness (Odawara et al., 2014).

For over a century, glial cells have been regarded as merely passive glue that connects and supports neurons. However, in recent years, they have been found to have diverse roles within the central nervous system (CNS). In particular, astrocytes, the most abundant cell type in the human CNS, are considered to be directly involved in brain signaling via astrocyte–neuron interaction at tripartite synapse (Perea et al., 2009; Bindocci et al., 2017; Martin-Fernandez et al., 2017). Recently, it has been proposed that astrocytes contribute to the progression of various neurodegenerative disorders such as Alzheimer’s, Parkinson’s, and Alexander’s disease (Brenner et al., 2001; Liddelow et al., 2017). Moreover, neurons and astrocytes have been reported to be closely involved in the onset of epileptic seizures (Onodera et al., 2021; Sano et al., 2021). Furthermore, *in vivo* experiments in rats demonstrated that antiepileptic drugs act on astrocytes, which may be related to their antiepileptic actions (Mukai et al., 2018).

From an electrophysiological perspective, epilepsy studies have indicated that wide-band electroencephalogram (EEG) activity in epileptic patients may reflect glial and neural activity. Ikeda et al. (2020) reported that recent improvements in digital electroencephalography have revealed that direct current (DC) potentials, which indicate activity in the low-frequency band below 1 Hz, reflect astrocyte-derived depolarization, whereas high-frequency oscillations (HFOs), which indicate activity in the high-frequency band above 200 Hz, reflect neuron firing activity. On the other hand, Fleischer et al. (2015) have shown that rat primary astrocytes respond to electrical stimulation with HFO. The relationship between DC potentials and HFO remains uncertain. Further studies at the cellular level are required in the future.

Most current MEA measurements focus on the spiking component of neurons in the frequency band above 50 Hz. In recent years, studies focusing on low-frequency components other than the spike component have been reported in MEA analysis of neural activity (Odawara et al., 2018; Trujillo et al., 2019; Yokoi et al., 2021; Sharf et al., 2022). To date, no MEA method has been established to measure glial-derived DC potentials in the lower-frequency band. In this study, we established a new measurement and analysis method focusing on DC potentials and report the first recording of spontaneous slow oscillatory activity of human cultured astrocytes using MEA. Furthermore, we demonstrated that this analysis method could be used to evaluate astrocyte drug responses to seizurogenic compounds.

## Materials and methods

### Culture of human astrocytes

Human primary astrocytes (Gibco) were cultured at 5.0 × 10^5^ cells/cm^2^ on 16 channels per well across 24-well MEA plates (MED-Q2430M; Alpha Med Scientific Inc.) coated with Geltrex (Gibco). Cells were maintained in astrocyte medium: Dulbecco’s modified Eagle medium (DMEM; Gibco) containing 10% fetal bovine serum (FBS; Gibco) and N-2 Supplement (Gibco). For culture on MEAs, a ϕ3.4-mm glass ring was placed in the middle of the MEA probe at the location of the electrode array, and cells were seeded inside the ring. After 1 h, astrocyte medium was added around the ring, and the ring was carefully removed. A frozen vial of human iPSC-derived mature astrocytes purchased from XCell Science was plated at 2.5 × 10^4^ cells/cm^2^ in tissue culture plates coated with Geltrex in STEMdiff Astrocyte Maturation Basal Medium kit (STEMCELL technologies). After 7 days, the astrocytes were sub-cultured at 5.0 × 10^5^ cells/cm^2^ on 24-well MEA plates in the same manner as above. All cells were maintained in astrocyte medium, and half the medium was replaced after 3 days.

### Immunocytochemistry

Sample cultures were fixed with 4% paraformaldehyde in phosphate-buffered saline (PBS) on ice (4°C) for 10 min. Fixed cells were incubated with 0.2% Triton X-100 in PBS for 5 min, followed by preblock buffer (0.05% Triton X and 5% FBS in PBS) at 4°C for 1 h. Samples were then incubated overnight at 4°C with primary antibodies diluted in preblock buffer. Primary antibodies used were glial fibrillary acidic protein (GFAP; 1:100; #3670, Cell Signaling Technology), human nuclei (1:100; MAB1281, Sigma-Aldrich), and MAP2 (1:1000; AB5622, Sigma-Aldrich). After being washed with PBS, samples were incubated with secondary antibodies, Alexa 488- or Alexa 546-conjugated anti-mouse IgG, anti-rabbit IgG (Invitrogen) for 30 min at room temperature. Cell nuclei were counterstained with 1 μg/mL Hoechst 33258 (H341, DOJINDO). All images were taken using an A1 Nikon confocal microscope system (Nikon). Image intensity was adjusted using ImageJ software (NIH).

### MEA measurements

This study requires the sensitivity of MEA to analyze signals in the frequency range below 1 Hz. Therefore, we used the MEA systems of Alpha Med Scientific Inc., which are excellent at detecting low-frequency components. Extracellular field potentials were measured using the 24-well MEA system (Presto) with a 20-kHz/channel sampling rate at 37°C under 5% CO_2_. The MEA system is implemented with a 0.1 Hz two pole Butterworth high-pass filter and a 5 kHz single pole Butterworth low-pass filter. 16 electrodes per well are arranged in a 4 × 4 array, with each electrode measuring 50 μm × 50 μm and spaced at 300 μm. All MEA measurements were performed in the astrocyte cell culture medium.

### Pharmacological test

After 1 week of culture, five seizurogenic compounds and two neutral compounds were cumulatively administered to human astrocytes. The following compounds were used as seizurogenic drugs: the potassium channel blocker 4-aminopyridine (4-AP; 0.3, 1, 3, 10, and 30 μM: 275875-1G, Sigma-Aldrich), the muscarinic receptor agonist pilocarpine (0.3, 1, 3, 10, and 30 μM: P6503-5G, Sigma-Aldrich), the GABAA receptor antagonist picrotoxin (0.1, 0.3, 1, 3, and 10 μM: P1675-1G, Sigma-Aldrich), 1,5-pentamethylene-tetrazole (PTZ; 30, 100, 300, 1,000, and 3,000 μM: P0046, Tokyo Chemical Industry Co.), and the D2 receptor antagonist chlorpromazine (0.1, 0.3, 1, 3, and 10 μM: C8138-5G, Sigma-Aldrich). Acetaminophen (1, 3, 10, 30, and 100 μM: A7085-100G, Sigma-Aldrich) and DMSO (D2650, Sigma-Aldrich) were used as neutral controls. VU0134992 (0.3, 1, 3, 10, 30 μM: 6877, Tocris Bioscience) and cilnidipine (1, 3, 10, 30, 100 μM: C2564, Tokyo Chemical Industry Co.) were used for blockage of Kir4.1 and calcium channels. The final concentrations of these drugs were adjusted to contain 0.1% DMSO. DMSO (0.1%) was administered in all wells as a vehicle control prior to cumulative administration of the compound. Spontaneous activities were recorded for 10 min after 5 min of rest following drug administration (*n* ≥ 3).

### Analysis parameters

The electrophysiological activity of astrocytes was analyzed by using Presto and Multi-bandpass Analysis software (Alpha Med Scientific). To separate the astrocyte activity to each frequency band, two pole Butterworth high-pass and two pole Bessel low-pass filters were used (DC, 0.1–1 Hz; delta, 1–4 Hz; theta, 4–8 Hz; alpha, 8–14 Hz; beta, 15–30 Hz; gamma, 35–50 Hz; high gamma, 80–150 Hz). The waveforms in each frequency band were converted into root mean square (RMS) histograms, and the threshold value was set at 10% of the highest potential value obtained during the total measurement time in 16 electrodes per well. RMS was calculated with a time window of 100 ms and shifted by 10 ms. Therefore, 90 ms was calculated by overlapping. We established nine analytical parameters for the RMS above the threshold value to evaluate the responses to compound: average total RMS, peak potential RMS, oscillation potential (OP), oscillation width (OW), coefficient of variation (CV) of OW, total OW, total oscillations, interpeak interval (IPI), and CV of IPI (Table 1).

**TABLE 1:** Description of analytical parameters.

Analytical parameter	Description
Average total RMS	Average of RMS calculated in 10 min
Peak potential RMS	Average peak value of RMS for all oscillations
Oscillation potential (OP)	Average of the sum of RMS values per bin for all oscillations
Oscillation width (OW)	Average duration of all oscillations
CV of OW	Coefficient of variation of total oscillation duration
Total OW	Sum of total duration of all oscillations
Total oscillations	The total number of all oscillations in 10 min
Inter peak interval (IPI)	Average time between peaks of all oscillations
CV of IPI	Coefficient of variation of time between peaks for all oscillations

Nine analytical parameters were calculated by multi-bandpass analysis.

### Frequency analysis

Power spectral analysis using Short-time Fourier transform (STFT) was performed on the base activity downsampled to 1kHz of primary astrocytes, no cells, fibroblasts, and DMSO 10% treated. A 20 s sliding temporal Hanning window was used in the spectral analysis and spectral power was calculated every 50 ms. To calculate the oscillation intensity without baseline effects, the moving sum of 400 points of the power spectrum was calculated, the minimum sum was defined as the baseline power, and the maximum sum of the spectrum minus the baseline power was calculated as the oscillation power.

### Statistical analysis

All data are representative of at least three independent sets of experiments. Analyses were conducted using GraphPad Prism software and the statistical software package R (http://www.r-project.org/). One-way ANOVA followed by Dunnett’s test was used to calculate the significant difference between each concentration and the vehicle. Statistical significance is indicated by the following: *p < 0.05, ^**^p < 0.01. Results are presented as mean ± SEM.

### Principal component analysis

We prepared a matrix using all well data (seizurogenic compounds: 4-AP, pilocarpine, picrotoxin, PTZ, and chlorpromazine; neutral controls: DMSO and acetaminophen) with nine analytical parameters obtained using the RMS histogram of DC- and delta-band activity. We performed principal component analysis (PCA) on 502 parameter sets to select two or more from nine analytical parameters using the MATLAB function PCA as previously reported (Ishibashi et al., 2021). One-way MANOVA was used to calculate the significant difference between each concentration and the vehicle in the first two principal components. We identified several parameter sets with significant differences between seizurogenic compounds and neutral compounds as effective parameter sets for detecting drug responses.

## Results

### Spontaneous activity of human astrocytes detected by MEA measurements

**FIGURE 1:**
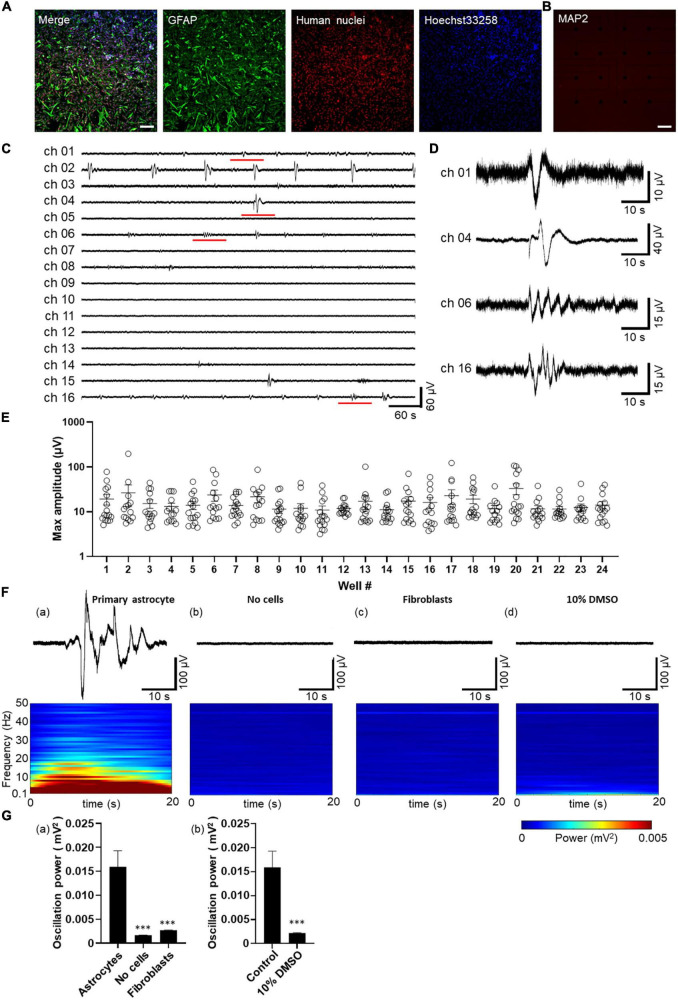
Spontaneous activity in human primary astrocytes detected by MEA. **(A,B)** Immunofluorescent images of astrocytes cultured on MEA at 8 days *in vitro* (8 DIV). Immunocytochemistry of GFAP (green), human nuclei (red), cell nuclei by Hoechst 33,258 (blue), merged images in *A*, and MAP2 (red) in **(B)** Scale bar = 200 μm. **(C)** Representative oscillation waveform at the spontaneous activity measurement for 10 min at 7 DIV. **(D)** The magnified waveform of the red underlined time in **(C)**. **(E)** Plot of maximum amplitude in 10 min oscillation waveform of each well. Error bars indicate the SEM. **(F)** Waveforms (upper panel) and their power spectrogram (lower panel) at maximum spectral intensity. (a) astrocytes, (b) no cells, (c) fibroblasts, (d) after 10% DMSO treatment. The vertical axis of the spectrogram shows the linear frequency from 0.1 Hz to 50 Hz, and the color indicates power. A 20 s spectrogram was extracted from the 40 s waveform. (G) Oscillation power of the control experiments. (a) Human primary astrocytes, no cells, fibroblasts, and (b) human primary astrocytes before and after 10% DMSO treatment. unpaired two-tailed *t*-tests, ^***^p < 0.001 versus astrocytes. Error bar, SEM.

Immunocytochemical staining was performed to confirm that the cultured cell samples on MEA were human astrocytes. Immunostaining with GFAP, a marker for astrocytes, and human nuclei, a marker for nuclei of all human cell types, indicated that the cells were human astrocytes (Figure 1A and Supplementary Figure 1A). To determine whether these cell populations contained neurons, the cells were stained with MAP2, a marker for mature neurons. No MAP2-positive cells were found in the cultured cells, confirming that astrocytes on MEA did not contain neurons (Figure 1B and Supplementary Figure 1B).

Spontaneous activity of human primary astrocytes was measured 7 days after the culture on MEA plates. Figures 1C, D show representative oscillation waveforms of spontaneous activity of astrocytes. To characterize the astrocyte activity obtained from MEA measurements, the acquired voltage waveforms were low-pass filtered at 50 Hz, and the maximum amplitude in 10 min for each electrode was calculated (n = 24 wells/360 electrodes). Figure 1E shows a plot of the maximum amplitude for each well. The minimum and maximum amplitude values of the electrodes at which oscillations were observed were 8.36 and 196.6 μV, respectively. We also observed spontaneous activity of human iPSC-derived astrocytes and compared to the primary astrocytes (Supplementary Figures 1C, D). The minimum and maximum amplitude values of the electrodes at which oscillations were observed in iPSC-derived astrocytes were 4.45 and 213.0 μV, respectively (Supplementary Figure 1E). Slow oscillatory activity was detected in all wells for human primary astrocyte, and 97% (33/34 wells) for iPS cell-derived astrocytes. The percentage of electrodes per well in which oscillatory activity was detected was 59.4% ± 12.5% (*n* = 24 wells) for human primary astrocyte and 26.7% ± 2.5% (*n* = 34 wells) for iPSC-derived astrocytes.

Next, to ensure whether the waveforms obtained reflected spontaneous activity derived from astrocytes, we performed several control experiments. To examine artifacts caused by electronics, we recorded from MEA covered with culture medium without cells for 3 weeks. Power spectral analysis using STFT was performed on the base activity of primary astrocytes and control samples. Figure 1F showed the waveform for the 40-second period of highest oscillation power (upper panel) and the power scalogram calculated from that waveform using STFT (lower panel). The frequency spectrum of astrocyte oscillations showed an intensity distribution in the 0.1 Hz to 10 Hz band [Figure 1F(a)]. In contrast, electrode not seeded with cells were observed every 7 days up to 3 weeks, but no oscillation was observed [Figure 1F(b)] and mean value and SEM of oscillation power was 0.016 ± 0.003 mV^2^ for astrocytes compared to 0.002 ± 0.00003 mV^2^ for the medium alone. [Figure 1G(a); n = 6 wells/96 electrodes; ^***^p < 0.001 versus astrocytes; unpaired two-tailed t-tests]. To test for astrocyte specificity, human fibroblasts were seeded on MEA at high density to cover the electrodes with cells. We observed up to 3 weeks, and any oscillation were not detected [Figure 1F(c)]. The oscillation power of fibroblasts was 0.003 ± 0.00008 mV^2^ [Figure 1G(a); *n* = 6 wells/96 electrodes; ^***^p < 0.001 versus astrocytes; unpaired two-tailed *t*-tests]. Finally, to exclude that a layer of biological materials of dead cells could cause the signals, primary astrocytes cultured on MEA were treated by 10% DMSO for killing cells. The oscillation was recorded before and 2 h after addition of DMSO. No oscillations were observed after the treatment [Figure 1F(d)] and DMSO addition significantly decreased oscillation power from 0.016 ± 0.003 mV^2^ to 0.002 ± 0.00007 mV^2^ [Figure 1G(b)]; n = 6 wells/96 electrodes; ^***^p < 0.001 versus astrocytes; unpaired two-tailed *t*-tests]. These results indicated that the slow oscillations observed by MEA are spontaneous activity of astrocytes on the electrode.

### Analytical parameters of spontaneous activity in human primary astrocytes

**FIGURE 2:**
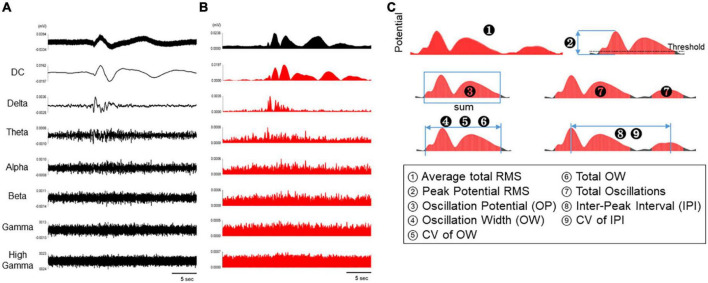
Analytical parameters of spontaneous activity in human primary astrocytes. **(A)** Representative oscillation waveform of spontaneous activity in human primary astrocytes at 3 DIV (top), and waveform separated into seven frequency bands (DC, 0.1–1 Hz; delta, 1–4 Hz; theta, 4–8 Hz; alpha, 8–14 Hz; beta, 15–30 Hz; gamma, 35–50 Hz; high gamma, 80–150 Hz). **(B)** The waveforms in each frequency band were converted into RMS histograms. **(C)** Schematic diagram of analysis parameters. The red area are subject to analysis for each parameter.

To assess astrocyte activity, the spontaneous activity of astrocytes was divided into the following frequency bands: DC potentials (0.1–1 Hz), delta (1–4 Hz), theta (4–8 Hz), alpha (8–14 Hz), beta (15–30 Hz), gamma (35–50 Hz), and high gamma (80–150 Hz) (Figure 2A). In addition, the waveforms in each frequency band were converted into RMS histograms in Figure 2B. The RMS histograms show that the slow waveforms of astrocyte spontaneous activity observed in the MEA measurements were most clearly reflected in DC potentials below 1 Hz, as reported in previous studies using electroencephalographs (Figure 2B). In contrast, astrocyte activity was not observed in the frequency bands higher than delta band (Figure 2B). Thus, the RMS of DC potential and delta band were used to analyze astrocyte activity. The threshold value was set at 10% of the highest potential value in all electrodes per well, and nine parameters were set for RMS above the threshold value: average total RMS, peak potential RMS, OP, OW, CV of OW, total OW, total oscillations, IPI, and CV of IPI (Figure 2C and Table 1).

### Astrocyte activity depends on ion channel modulations

**FIGURE 3:**
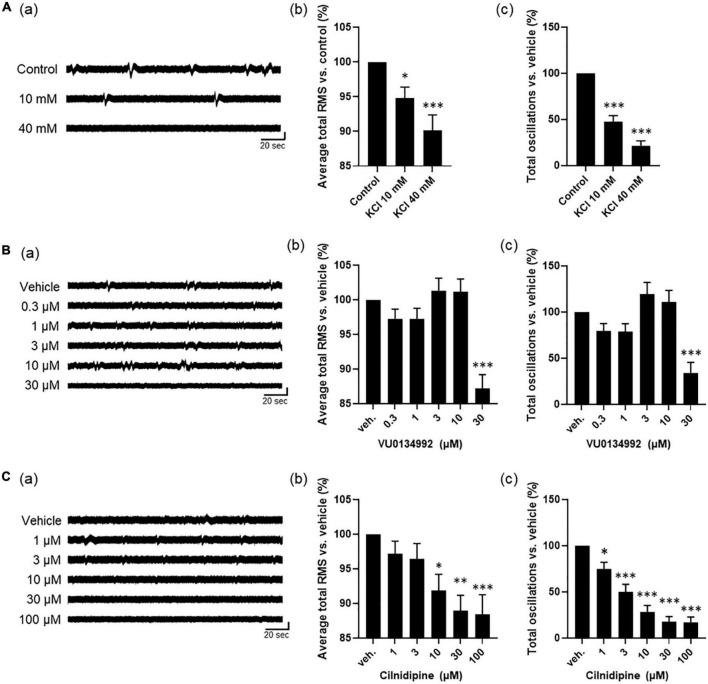
Astrocyte activity depends on ion channel modulations. **(A)** high potassium experiments by cumulative administration of KCl (*n* = 5). (a) Representative oscillation waveform. (b) Average total RMS and (c) Total oscillations. **(B)** Inhibitory experiment by VU0134992 (*n* = 6). (a) Representative oscillation waveform. (b) Average total RMS and (c) Total oscillations. **(C)** Inhibitory experiment by cilnidipine (*n* = 4). (a) Representative oscillation waveform. (b) Average total RMS and (c) Total oscillations. KCl and inhibitors were added to astrocytes at 7 DIV. One-way ANOVA followed by Dunnett’s test, *p < 0.05, ^**^p < 0.01, ^***^p < 0.001 versus control/vehicle. Error bar, SEM.

We next explored a mechanism of the astrocyte spontaneous slow oscillatory activity by using human primary astrocytes. Ikeda et al. reported that DC potential observed in wide-band EEG activity of epileptic patients associated to extracellular potassium, and hypothesized the potassium homeostasis are relative to regulation by Kir4.1 channels in astrocytes. Therefore, we first investigated the effect of extracellular potassium concentration on astrocyte slow oscillations. Based on the chemical composition of ACSF, which maintains comparable electrolyte concentration as cerebrospinal fluid, the extracellular potassium concentration at physiological levels is considered to be 3.0 mM. On the other hand Ullah et al. (2009) measured extracellular neuro-glial potassium concentrations and reported that the extracellular potassium concentration at epileptogenic levels was approximately 40 mM at peak values. They also reported that neuronal firing is induced from an extracellular potassium concentration of 8.5 mM. Medium used for MEA measurements in this study contained 5.3 mM of KCl, and its potassium concentration is close to physiological levels compared to epileptogenic levels and do not reach concentrations that would induce neuronal firing. Therefore, we treated human primary astrocytes with KCl 10 mM, the concentration at which nerve firing can be induced, and KCl 40 mM, the epileptogenic level, and observed changes in oscillation in DC potential. The oscillation frequency decreased with increasing KCl concentrations [Figure 3A(a)]. To analyze the effect on astrocyte activity, we examined two parameters: average total RMS, which indicates the intensity of oscillations, and total oscillations, which reflects the frequency of oscillations. A dose-dependent decrease in these two parameters was observed [Figures 3A(b, c)]; *n* = 5 wells; *p < 0.05, ^***^p < 0.001 versus control; one-way ANOVA followed by Dunnett’s test]. We next investigated whether regulation of extracellular potassium concentrations by Kir4.1 channels was involved in the formation of astrocyte slow oscillations. The results showed loss of oscillation at 30 μM with 100% inhibitory effect [Figure 3B(a)]. We also found significant reductions at 30 μM in average total RMS and total oscillations [Figures 3B(b, c); *n* = 6 wells; ^***^p < 0.001 versus vehicle; one-way ANOVA followed by Dunnett’s test]. These results indicate that extracellular potassium concentration through Kir4.1 channels is involved in slow oscillation formation in astrocytes. Finally, to confirm the effect of calcium, which plays an important role in astrocyte function, we performed experiments with the calcium channel inhibitor cilnidipine. Cilnidipine showed reduced astrocyte slow oscillations in dose-dependent manner and also indicated in average total RMS and in total oscillations (Figure 3C; *n* = 4 wells; *p < 0.05, ^**^p < 0.01, ^***^p < 0.001 versus vehicle; one-way ANOVA followed by Dunnett’s test). These results suggested that calcium regulation plays an important role in the formation of slow oscillations in astrocytes.

### Drug response of human primary astrocytes in MEA

**FIGURE 4:**
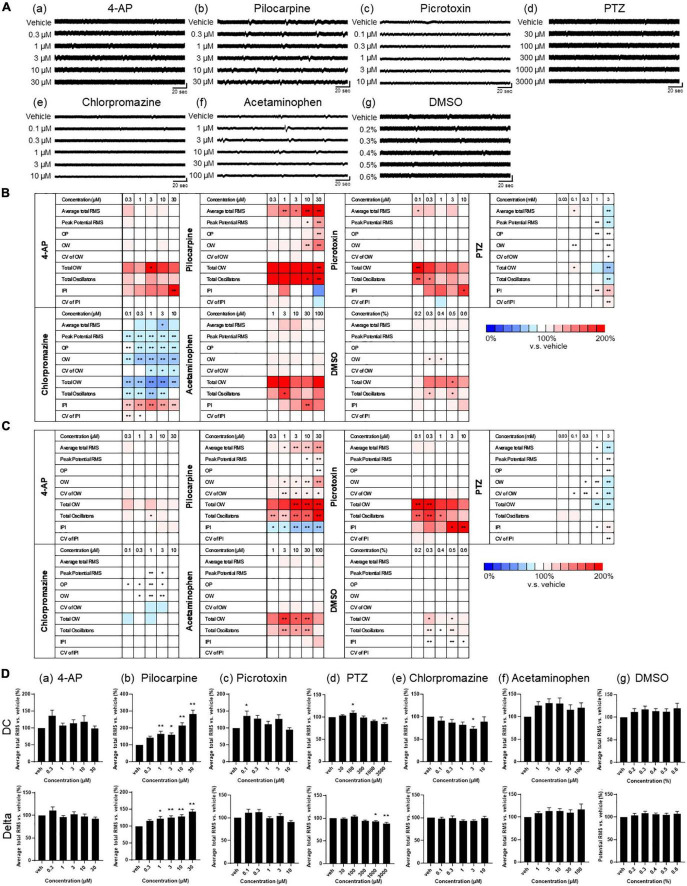
Drug responses of primary astrocytes detected by MEA. **(A)** Representative oscillation waveform after the cumulative administration of seizurogenic compounds (a) 4-AP, (b) pilocarpine, (c) picrotoxin, (d) PTZ, and (e) chlorpromazine, and neutral compounds (f) acetaminophen and (g) DMSO. Compounds were added to astrocytes during 1–3 weeks of the culture. Vertical scale bar, 40 μV; horizontal scale bar, 20 ms. **(B,C)** Heatmaps of the analytical parameters of seizurogenic compounds and neutral compounds in DC potential **(B)** and in delta band **(C)**. 4-AP (*n* = 6), pilocarpine (*n* = 6), picrotoxin (*n* = 6), PTZ (*n* = 6), chlorpromazine (*n* = 6), acetaminophen (*n* = 6), and DMSO (*n* = 6). **(D)** Dose-dependent changes of average total RMS in DC potential (upper) and in delta band (lower). One-way ANOVA followed by Dunnett’s test, *p < 0.05, **p < 0.01 versus vehicle. Error bar, SEM.

We assessed the drug responses of astrocytes during 1–3 weeks of culture using nine analytical parameters. Five representative seizurogenic compounds, namely, 4-AP, pilocarpine, picrotoxin, PTZ, and chlorpromazine, were added to the medium of astrocytes cultured on MEA, and the responses of astrocytes against these compounds were examined. Acetaminophen and DMSO responses were used as neutral controls. Figure 4A represents the astrocyte waveforms for each compound. Among the five seizurogenic compounds, the most significant change was observed with the muscarinic receptor agonist pilocarpine, which increased the oscillation frequency on the waveform in a dose-dependent manner [Figure 4A(b)]. Figures 4B, C show the analysis results of the compound responses using nine analysis parameters in DC potential and delta band, respectively (*n* = 6 wells for each compound; *p < 0.05, ^**^p < 0.01 versus vehicle; one-way ANOVA followed by Dunnett’s test). The rate of change and its significance were indicated when each sample with vehicle only was defined to be 100%. Among those analytical parameters, the average total RMS is graphed in Figure 4D. Pilocarpine administration lead to significantly elevated average total RMS of the DC potential at concentrations above 1 μM [Figures 4B, D(b)]. Pilocarpine also showed some significant increases in other parameters at high concentrations (Figure 4B). Picrotoxin, a GABAA receptor antagonist, showed the highest frequency of oscillations at 0.1 μM, with significant increases in several parameters, including average total RMS [Figures 4A(c), B, D(c)]. With PTZ treatment, the average total RMS increased at 100 μM and showed a dose-dependent decrease with subsequent concentrations [Figures 4A(d), B, D(d)]. Chlorpromazine, a D2 receptor antagonist, showed reduced astrocyte oscillations, with a significant decrease at 3 μM in average total RMS [Figures 4A(e), B, D(e)]. Unlike the other seizurogenic compounds, chlorpromazine demonstrated significant decreases for many parameters. Interestingly, the K-ion channel blocker 4-AP, a typical seizurogenic compound, showed no significant changes, although some parameters tended to increase [Figures 4A](a), B, D(a)]. Although the analytical parameters that showed changes differed among the compounds, the 4-AP results were similar to those of the neutral controls, acetaminophen and DMSO, which showed less significant changes [Figures 4A(f, g), B, D(f, g)].

Analysis of the delta frequency band showed a dose-dependent increase only for pilocarpine in average total RMS (Figures 4C, D; *n* = 6 wells for each compound; *p < 0.05, ^**^p < 0.01 versus vehicle; one-way ANOVA followed by Dunnett’s test). For the other parameters, there were slightly different parameters and concentrations that showed significant changes for each compound, but the tendency of increase or decrease was similar to the results of the analysis of DC potentials (Figure 4C).

### PCA of drug response in human primary astrocytes

**FIGURE 5:**
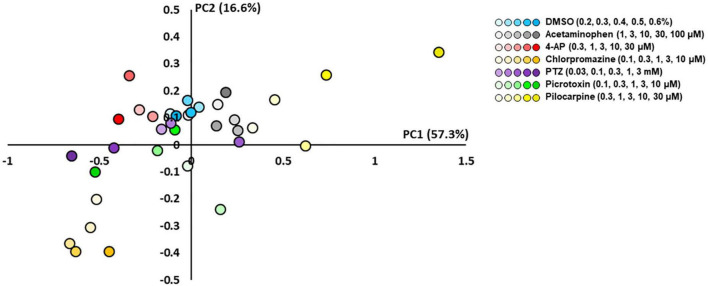
Scatterplots of principal component analysis (PCA) using the effective parameter set for detecting the drug responses of primary astrocytes. There was a clear separation between the seizurogenic and neutral compounds and between all seizurogenic compounds. In neutral compounds, DMSO and acetaminophen were not separated. DMSO (*n* = 6, blue), acetaminophen (*n* = 6, gray), 4-AP (*n* = 6, red), chlorpromazine (*n* = 6, orange), PTZ (*n* = 6, purple), picrotoxin (*n* = 6, green), and pilocarpine (*n* = 6, yellow). Higher concentrations are indicated by darker colored symbols.

To examine whether the response of astrocytes to seizurogenic and neutral controls can be separated, a PCA was performed using the nine parameters in DC potential and delta band. Using one-way MANOVA, we identified five parameters that showed no significant differences between neutral controls in PC1 and PC2 and displayed significant differences between seizurogenic and neutral controls and between all seizurogenic compounds (Table 2). Figure 5 shows the results of the PCA with the identified parameters. No significant differences were found between the neutral compounds DMSO and acetaminophen, and significant differences of *p* < 0.05 were detected between the seizurogenic and neutral compounds and between all seizurogenic compounds (Table 3). The cumulative contribution of PC1 and PC2 was 73.9%. These results indicate that astrocyte responses to seizurogenic and neutral compounds can be separated by MEA measurements. Furthermore, since the response to each seizurogenic compound was also separable, it was suggested that the mechanism of action of each drug could also be separated by MEA measurement. The most pronounced changes, pilocarpine and chlorpromazine, were at a greater distance from the neutral controls, with pilocarpine exhibiting a dose-dependent change in the upper right diagonal of the PC1–PC2 plot and chlorpromazine in the lower left diagonal. Picrotoxin and PTZ showed a dose-dependent PC1 shift in a negative (leftward) direction, whereas 4-AP showed a slightly upper left diagonal shift. It is suggested that the present results reflect differences in astrocyte drug response (Figure 5).

**TABLE 2:** Principal component loadings for PCA using effective parameter set for detecting the drug responses in human primary astrocytes.

	Principal component loadings	
**Parameter**	**PC1**	**PC2**
OW_DC	0.31	0.86
Average total RMS_Delta	0.43	−0.31
Peak potential RMS_Delta	0.56	−0.02
OP_Delta	0.54	0.01
Total oscillations_Delta	0.34	−0.40

**TABLE 3:** Statistical analysis for PCA using effective parameter set for detecting the drug responses in human primary astrocytes.

	vs. DMSO	vs. Acetaminophen	vs. 4-AP	vs. Chlorpromazine	vs. PTZ	vs. Picrotoxin	vs. Pilocarpine
**Compounds**	***p* value**	***p* value**	***p* value**	***p* value**	***p* value**	***p* value**	***p* value**
DMSO	—	*p* = 0.204	*p = 0.014	**p = 3.71e-71	**p = 1.31e-05	**p = 1.28e-04	**p = 4.33e-17
Acetaminophen	*p* = 0.204	—	**p = 1.27e-05	**p = 2.99e-33	**p = 1.79e-03	**p = 1.13e-04	**p = 1.46e-06
4-AP	*p = 0.014	**p = 1.27e-05	—	**p = 8.64e-33	**p = 7.72e-03	**p = 1.50e-03	**p = 1.05e-21
Chlorpromazine	**p = 3.71e-71	**p = 2.99e-33	**p = 8.64e-33	—	**p = 2.78e-66	**p = 8.57e-13	**p = 2.03e-56
PTZ	**p = 1.31e-05	**p = 1.79e-03	**p = 7.72e-03	**p = 2.78e-66	—	*p = 0.015	**p = 5.33e-11
Picrotoxin	**p = 1.28e-04	**p = 1.13e-04	**p = 1.50e-03	**p = 8.57e-13	*p = 0.015	—	**p = 5.97e-15
Pilocarpine	**p = 4.33e-17	**p = 1.46e-06	**p = 1.05e-21	**p = 2.03e-56	**p = 5.33e-11	**p = 5.97e-15	—

4-AP (*n* = 6), pilocarpine (*n* = 6), picrotoxin (*n* = 6), PTZ (*n* = 6), chlorpromazine (*n* = 6), acetaminophen (*n* = 6), and DMSO (*n* = 6). One-way MANOVA. *p < 0.05, **p < 0.01.

### Drug response of human iPSC-derived astrocytes in MEA

**FIGURE 6:**
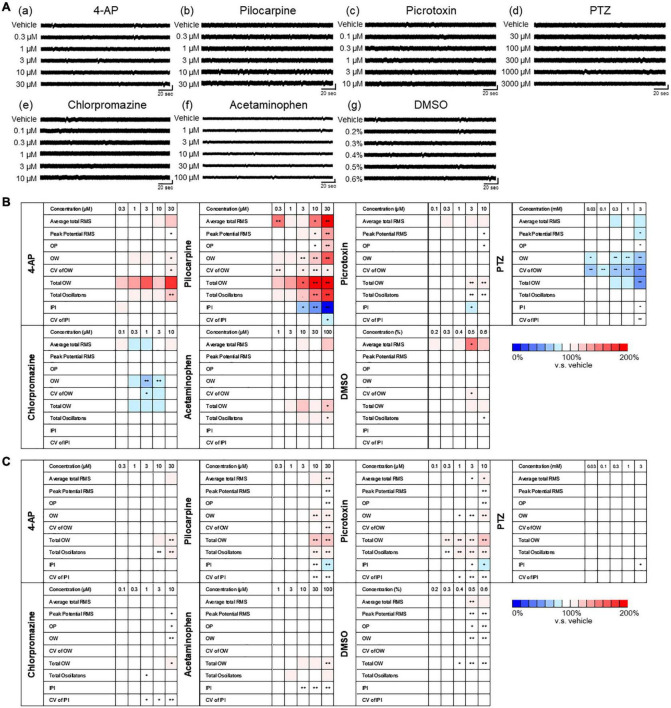
Drug responses of human iPSC-derived astrocytes detected by MEA. **(A)** Representative oscillation waveform after the cumulative administration of seizurogenic compounds (a) 4-AP, (b) pilocarpine, (c) picrotoxin, (d) PTZ, and (e) chlorpromazine, and neutral compounds (f) acetaminophen and (g) DMSO. Compounds were added to astrocytes during 2–3 weeks of the culture. Vertical scale bar, 40 μV; horizontal scale bar, 20 ms. **(B,C)** Heatmaps of the analytical parameters of seizurogenic compounds (4-AP, *n* = 6; pilocarpine, *n* = 6; picrotoxin, *n* = 6; chlorpromazine, *n* = 6) and neutral compounds (acetaminophen, *n* = 6; DMSO, *n* = 6) in DC potential **(A)** and in delta band **(B)**. One-way ANOVA followed by Dunnett’s test, *p < 0.05, **p < 0.01 versus vehicle.

Next, drug responses of human iPSC-derived astrocytes were examined and compared with those of human primary astrocytes. Five seizurogenic compounds with different mechanisms of action (4-AP, pilocarpine, picrotoxin, PTZ, and chlorpromazine) and two neutral controls (DMSO and acetaminophen) were added to human iPSC-derived astrocytes on MEA during 2–4 weeks of culture (Figure 6A), and astrocyte responses to each drug were evaluated using the nine analytical parameters. The rate of change and its significance of DC and delta bands were indicated when each sample with vehicle only was defined to be 100% (Figures 6B, C; *n* = 6 wells for each compound; *p < 0.05, ^**^p < 0.01 versus vehicle; one-way ANOVA followed by Dunnett’s test). Comparison of DC potentials and delta band indicated similar changes in both frequency bands, but DC potentials showed a greater rate of change. This suggests that DC potentials below 1 Hz in human iPSC-derived astrocytes as well as in human primary astrocytes more strongly reflect astrocyte responses to drugs. Among the five seizurogenic compounds, pilocarpine demonstrated the most significant changes. Although significant increases were observed at 0.3 μM for average total RMS and CV of OW for DC potential, pilocarpine showed dose-dependent changes in many parameters for both DC potential and delta band (Figures 6B, C). This result was similar to that of primary astrocytes (Figure 4B). Interestingly, significant increases were observed at concentrations higher than 30 μM for 4-AP and 3 μM for picrotoxin (Figures 6B, C). A significant decrease was also observed with PTZ and chlorpromazine compared to vehicle (Figure 6B). For the neutral compounds, there was a significant increase in average total RMS at 0.5% DMSO, but great increase or decrease was not found in both DC potential and delta band (Figures 6B, C). These results indicated that the rate of change was smaller in iPSC-derived astrocytes than in primary astrocytes. Although the concentrations at which significant changes were observed differed in some compounds from those in primary astrocytes, there was a similar trend of increase or decrease depending on the compound.

**FIGURE 7:**
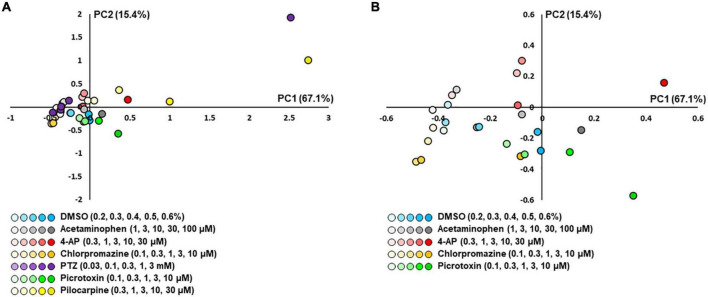
Scatterplots of PCA using the effective parameter set for detecting the drug responses of iPSC-derived astrocytes. **(A)** DMSO (*n* = 6, blue), acetaminophen (*n* = 6, gray), 4-AP (*n* = 6, red), chlorpromazine (*n* = 6, orange), PTZ (*n* = 6, purple), picrotoxin (*n* = 6, green), and pilocarpine (*n* = 6, yellow). Higher concentrations are indicated by darker colored symbols. **(B)** The magnified scatterplots excluding the plots of pilocarpine and PTZ.

We next performed a PCA using nine parameters to determine whether responses to the compounds could be separated in iPSC-derived astrocytes. Five parameters were identified that showed no significant differences between neutral compounds in PC1 and PC2, and significant differences between seizurogenic and neutral compounds and between all seizurogenic compounds (Table 4). Figure 7 is the results of a PCA using these parameters. No significant differences were observed between the neutral controls DMSO and acetaminophen; however, significant differences were observed between the seizurogenic and neutral controls, *p* < 0.01 (Table 5). There was also a significant difference between all seizurogenic compounds, *p* < 0.05 (Table 5). The cumulative contribution of PC1 and PC2 was 82.5%. The PCA result of human iPSC-derived astrocytes indicated that the largest change was observed in pilocarpine and 3 mM of PTZ (Figure 7). Similar to the results for human primary astrocytes, pilocarpine showed a dose-dependent change in the upper right diagonal of the PC1–PC2 plot. In PTZ, as same as primary astrocytes, the concentrations under 1 mM showed a dose-dependent PC1 shift in a negative direction, but 3 mM of PTZ showed a big change in the opposite direction. Figure 7B shows magnified scatterplots excluding the plots of pilocarpine and PTZ. In the principal component plot, the tendency for pilocarpine and chlorpromazine to increase their distances in the opposite direction was similar to that of primary astrocytes. Picrotoxin demonstrated a dose-dependent change in a different direction (lower right diagonal) from PTZ, chlorpromazine and pilocarpine. 4-AP showed a different distribution from the neutral compounds, although the percentage change was small. These results indicated that it is possible to separate responses to seizurogenic and neutral compounds in iPSC-derived astrocytes as well as in primary astrocytes and that it is also possible to separate responses to each seizurogenic compound.

**TABLE 4:** Principal component loadings for PCA using effective parameter set for detecting the drug responses in human iPSC-derived astrocytes.

	Principal component loadings	
**Parameter**	**PC1**	**PC2**
Peak potential RMS_DC	0.47	0.14
OW_DC	0.45	0.57
Total OW_DC	0.42	0.34
OW_Delta	0.45	−0.52
Total OW_Delta	0.44	−0.52

**TABLE 5:** Statistical analysis for PCA using effective parameter set for detecting the drug responses in human iPSC-derived astrocytes.

	vs. DMSO	vs. Acetaminophen	vs. 4-AP	vs. Chlorpromazine	vs. PTZ	vs. Picrotoxin	vs. Pilocarpine
**Compounds**	***p* value**	***p* value**	***p* value**	***p* value**	***p* value**	***p* value**	***p* value**
DMSO	—	*p* = 0.101	**p = 4.30e-06	**p = 8.50e-07	**p = 1.12e-11	** p = 7.16e-06	**p = 1.92e-21
Acetaminophen	*p* = 0.101	—	**p = 5.66e-3	**p = 2.35e-09	**p = 2.62e-07	** p = 3.26e-07	**p = 1.35e-17
4-AP	**p = 4.30e-06	**p = 5.66e-3	—	**p = 4.1e-09	*p = 0.032	** p = 5.09e-13	**p = 1.12e-07
Chlorpromazine	**p = 8.50e-07	**p = 2.35e-09	**p = 4.1e-09	—	**p = 2.13e-12	** p = 6.44e-06	**p = 1.94e-22
PTZ	**p = 1.12e-11	**p = 2.62e-07	*p = 0.032	**p = 2.13e-12	—	** p = 1.39e-19	**p = 6.47e-06
Picrotoxin	**p = 7.16e-06	**p = 3.26e-07	**p = 5.09e-13	**p = 6.44e-06	**p = 1.39e-19	—	**p = 7.88e-26
Pilocarpine	**p = 1.92e-21	**p = 1.35e-17	**p = 1.12e-07	**p = 1.94e-22	**p = 6.47e-06	**p = 7.88e-26	—

4-AP (*n* = 6), pilocarpine (*n* = 6), picrotoxin (*n* = 6), PTZ (*n* = 6), chlorpromazine (*n* = 6), acetaminophen (*n* = 6), and DMSO (*n* = 6). One-way MANOVA. *p < 0.05, **p < 0.01.

## Discussion

Since the first report of neural activity by Thomas et al. (1972) in, extracellular electrical activity has been recorded in various types of neurons and neural tissues (Thomas et al., 1972; Johnstone et al., 2010). However, to the best of our knowledge, there have been no reports of extracellular electrical activity recorded in human astrocyte-only culture samples using MEA. In this study, we have succeeded in measuring the slow oscillatory activity of cultured astrocytes using MEA. Furthermore, we established a method for assessing astrocyte activity using nine analytical parameters and demonstrated that drug responses to five seizurogenic compounds could be separated by PCA.

In this study, we used two types of commercially available astrocytes, human primary astrocytes and human iPSC-derived astrocytes, and measured their spontaneous activity and responses to the drugs. For spontaneous activity, compared to primary astrocytes, the percentage of oscillations detected was lower in human iPSC-derived astrocytes. The average maximum voltage of oscillation at each electrode was 25.51 ± 1.68 μV (*n* = 191 electrodes) for primary astrocytes and 22.72 ± 1.95 μV (*n* = 142 electrodes) for iPSC-derived astrocytes, *p* = 0.28. This might be due to the immaturity of human iPSC-derived astrocytes compared to primary astrocytes and the relationship between astrocyte maturation and signal acquisition is a future issue.

We demonstrated that extracellular potassium concentration is involved in generation of astrocyte slow oscillations observed in MEA (Figures 3A, B). In Ikeda et al.’s paper, one hypothesis is related to deficient potassium buffering capacity of astrocytes related to Kir4.1 channels and a high extracellular potassium concentration leading to epileptiform activity (Ikeda et al., 2020). However, astrocyte oscillations detected by MEA were significantly reduced in both intensity and frequency when treated with 40 mM KCl at epileptogenic levels (Figure 3A). On the other hand, this result was similar to that of Fleischer et al. who recorded stimulation-dependent astrocyte activity of HFOs using MEA (Fleischer et al., 2015). Regarding the effect of calcium modulation, the results of inhibition experiments with cilnidipine showed dose-dependent decrease in astrocyte oscillations, and it was consistent with those reported by Fleischer et al. (Figure 3C). In addition, many previous studies reported that intracellular Ca^2+^ concentration in astrocytes dynamically changed in response to drugs (Heuser and Enger, 2021). Therefore, changes in calcium concentration have a role in astrocyte oscillations observed in this study. For the baseline signal, we observed a heterogeneity of oscillations. The oscillations were found to be dependent on ion channels, but it is still difficult to define the influx of various ions from the shape of the waveform. The amplitude of the signal varies depending on the location of the electrodes and the cells. In addition, it is the sum of ion influxes from multiple channels. Therefore, we hypothesize that this is the reason for the heterogeneity of the waves. The physiological significance of waveforms and signal propagations are subjects for future study.

To examine astrocyte drug responses, five seizurogenic compounds were added to human primary astrocytes and human iPSC-derived astrocytes. The drug that showed the most significant response in both cells was pilocarpine, a muscarinic receptor agonist. (Figures 4, 6). Pilocarpine is one of the most widely used seizure-inducing compounds to prepare animal models of epilepsy in glia research. Astrocytes, as well as neurons, express muscarinic receptors (Murphy et al., 1986; Guizzetti et al., 1996), and our results of the dose-dependent increase in oscillation frequency indicated that pilocarpine affected muscarinic receptors in astrocytes. The EEG analysis in a rat model of epilepsy using pilocarpine has reported that the low-frequency component DC shift may reflect a disruption of spatial K^+^ interference function in astrocytes (Ikeda et al., 2020), suggesting that changes in K^+^ concentration may also be affected in the astrocyte oscillation activity observed in our DC potentials in this study.

Chlorpromazine, a D2 receptor antagonist, was observed to reduce oscillation activity in both primary astrocytes and iPSC-derived astrocytes (Figures 4, 6). D2 receptors are known to be expressed in astrocytes (Bal et al., 1994; Zanassi et al., 1999; Miyazaki et al., 2004), but the effects of chlorpromazine on astrocyte D2 receptors are not yet well understood. Chlorpromazine also acts as a calmodulin antagonist, which inhibits the catalytic activity of plasma membrane Ca^2+^-ATPase to prevent transfer of calcium ions from cytosol into the extracellular space (Khan et al., 2001; Plenge-Tellechea et al., 2018). Our results showing the negative change in astrocyte activity with chlorpromazine treatment suggested that the transfer of calcium ions might have been inhibited by chlorpromazine. Furthermore, Chu et al. (2022) reported that when human primary astrocytes were treated with chlorpromazine at concentrations from 10 to 50 μM for 24 h, cell death was observed at 50 μM, but [Ca^2+^]i measurements using a spectrophotometer with Fura-2-AM showed no change at 10–30 μM. Our MEA measurements successfully captured astrocyte responses at concentrations (0.1–10 μM) even lower than those concentrations.

Interestingly, although both picrotoxin and PTZ are GABAA receptor antagonists, the responses of astrocytes in this study to each drug showed different trends and were separated in PCA (Figures 5, 7). PTZ has been reported to inhibit the GABAA receptor by interacting with the picrotoxin-barbiturate binding site, closing Cl^–^ channels, and provoking seizures (Qu et al., 2005). Similarly, Fraser et al. (1995) have shown that treatment of astrocytes with picrotoxin inhibits GABAA-activated Cl^–^ conductance by using whole-cell patch clamping. These reports suggested that the astrocyte responses to picrotoxin and PTZ in this study involves changes in Cl ion concentration. However, PTZ has been reported to affect mitochondrial metabolism and glycolysis in cortical and cerebellar astrocytes in culture (Qu et al., 2005). Another group has demonstrated that mitochondria presenting within the fine processes of astrocytes contribute to local Ca^2+^ signaling within the astrocyte (Jackson and Robinson, 2018). These reports suggest that PTZ may be involved in Ca^2+^ signaling as well as Cl^–^ signaling. Experiments on zebrafish compared the effects of picrotoxin and PTZ and demonstrated that each drug has a different mechanism *in vivo* (Yang et al., 2017). Our PCA results demonstrated that the drug responses of PTZ and picrotoxin were separated in the primary and iPSC-derived astrocytes (Figures 5, 7). This indicated that our evaluation system assessed the subtle differences in the mechanism of action of each drug.

4-AP, a typical seizurogenic compound, showed a comparable response to neutral controls in both primary astrocytes and iPSC-derived astrocytes (Figures 4, 6). Recently, it has been reported that astrocytes have a protective function for neurons during seizures induced by 4-AP in neuron-astrocyte co-cultures (Ahtiainen et al., 2021). 4-AP is a voltage-gated K^+^ channel blocker. Astrocytes express voltage-gated K^+^ channels and non-voltage-gated K^+^ channels (e.g., Kir channels, Na^+^/K^+^-ATPase, and Na^+^/K^+^/2Cl^–^ cotransporters). Ahtiainen et al. (2021) suggested that the protective role of astrocytes under 4-AP treatment may have been due to the non-voltage-gated K^+^ channels in astrocytes that maintained K^+^ homeostasis. In the present study, it was possible that astrocytes may have maintained K^+^ via the non-voltage-gated K^+^ channel, which was not inhibited by 4-AP, resulting in no significant change in K^+^ and a response comparable to that of the negative compound. From these results of drug responses, we demonstrated that it is possible to measure astrocyte drug response using MEA. Our results of the drug response showed that astrocytes responded differently to each drug, suggesting that the oscillation pattern of astrocytes is a phenomenon caused by the balance of factors on which each drug acts, such as K^+^, Cl^–^, and Ca^2+^. The PCA in this study might have captured the changes in oscillation patterns resulted from those ion balance and successfully separated drug reactions (Figures 5, 7). However, the physiological relevance of each parameter and channel responses has not yet been understood. In order to develop MEA measurements of astrocytes, this is an important issue to be resolved in the future.

For drug responses, both astrocytes demonstrated the dose-dependent response to pilocarpine and showed the negative response to chlorpromazine. These results indicated that our MEA method for cultured astrocytes could assess drug responses even in astrocytes derived from different cell sources. However, slightly different responses were observed between the two cell types. Primary astrocytes responded more strongly to drugs than did human iPSC-derived astrocytes. These observed differences between primary astrocytes and iPSC-derived astrocytes may be due to astrocyte maturity, but they may also be due to the diversity of gene expression in astrocytes. Lundin et al. (2018) reported large diversity among astrocytic models derived from various sources using several analytical methods, including transcriptomic and proteomic analyses. The PCA results also suggested that primary astrocytes and iPSC-derived astrocytes have different properties, since the parameter sets of primary and iPSC-derived astrocytes were different. The parameters of the set showed that primary astrocytes had more delta components than iPSC-derived astrocytes. This suggests that different cells may have slightly different main frequency bands for astrocyte signals. The correlation between astrocyte diversity and the differences in drug response in each astrocyte in the MEA measurement should be evaluated in the future.

MEA is a measurement method with high temporal resolution and noninvasiveness that enables long-term electrical activity recording. In the current astrocyte research, two major methods are used to assess real-time astrocyte electrophysiological activity: one is the traditional patch clamp method, and the other is the Ca imaging, which has been used extensively in astrocyte research since the 1990s. In addition to these methods, the evaluation of extracellular potential activity of astrocytes using the MEA method proposed here is expected to enable more detailed analysis of astrocyte activity. In recent years, the involvement of glia in various neurological diseases has been reported, and studies using patient iPSC-derived astrocytes for Rett syndrome, Alexander’s disease, Alzheimer’s disease, and autism spectrum disorder, in which astrocytes are thought to be involved, are actively conducted (Williams et al., 2014; Kondo et al., 2016; Jones et al., 2017; Russo et al., 2018; Salcedo et al., 2021; Allen et al., 2022). We expect that the MEA-based system for assessing astrocyte activity will not only provide new insights into the mechanisms of neurological diseases, including epilepsy, but will also help in the evaluation of drug efficacy in the drug development for astrocyte related diseases.

## Data availability statement

The raw data supporting the conclusions of this article will be made available by the authors, without undue reservation.

## Author contributions

TK, NM, and IS designed the research. TK performed the research. TK, NM, and YI analyzed the data. TK and IS wrote the manuscript. All authors contributed to the article and approved the submitted version.

## Supplementary material

Supplementary Figure 1 Spontaneous activity in human iPSC-derived astrocytes detected by MEA. **(A,B)** Immunofluorescent images of astrocytes cultured on MEA at 35 DIV. Immunocytochemistry of GFAP (green), human nuclei (red), cell nuclei by Hoechst 33258 (blue), merged images in **(A)** and MAP2 (red) in **(B)**. Scale bar = 200 μm. **C** Representative oscillation waveform at the spontaneous activity measurement for 10 min at 14 DIV. **(D)** The magnified waveform of the red underlined time in **(C)**. **(E)** Plot of maximum amplitude in 10 min oscillation waveform of each well. Error bars indicate the SEM.
