# Supplementary figures and images for: Detection of astrocytic slow oscillatory activity and response to seizurogenic compounds using planar microelectrode array
Source: Front Neurosci. 2023 Jan 10;16:1050150. doi: 10.3389/fnins.2022.1050150 (PMC9872017; doi:10.3389/fnins.2022.1050150)

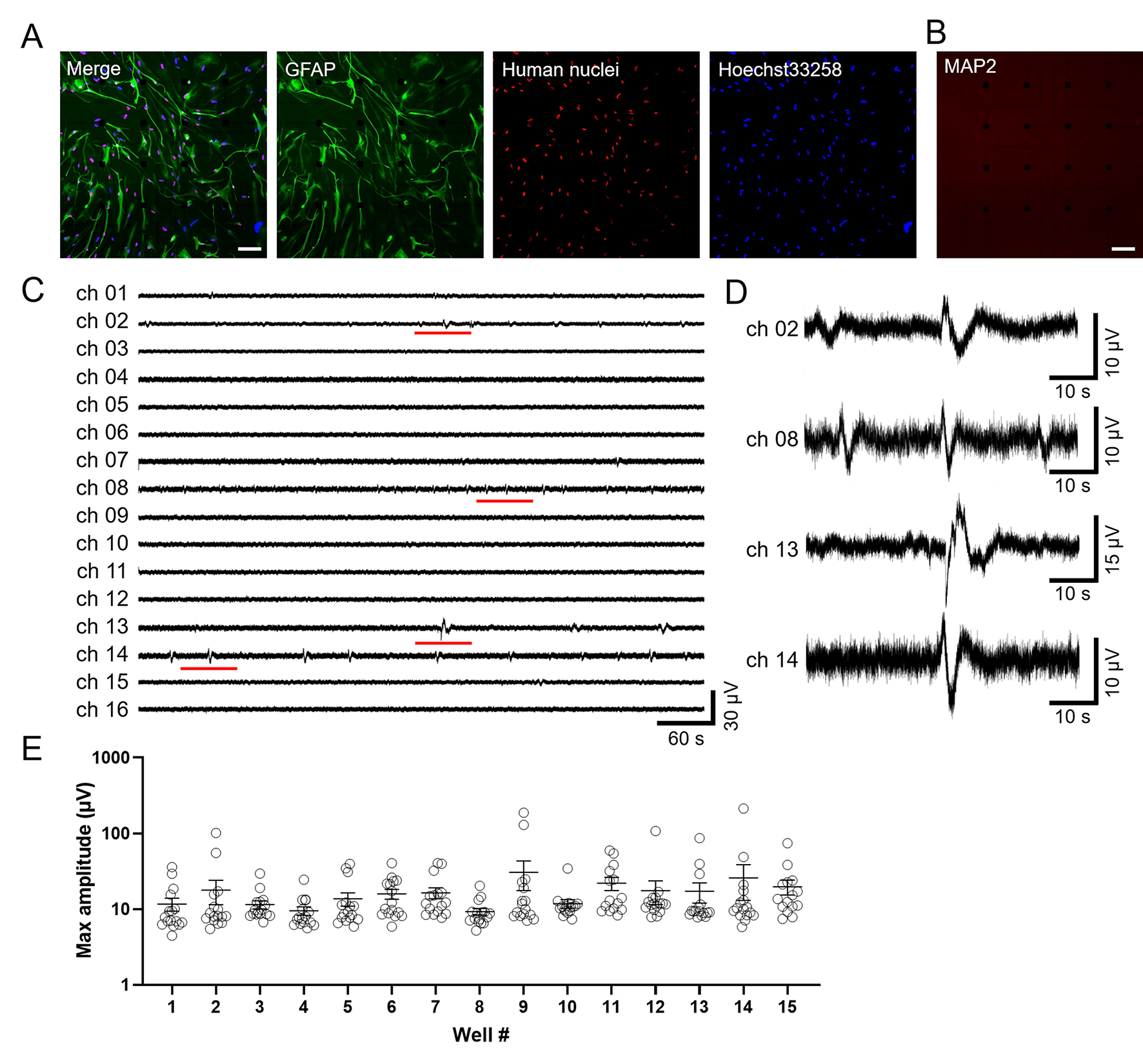

Supplement: Supplementary Figure 1 — Spontaneous activity in human iPSC-derived astrocytes detected by MEA. (A,B) Immunofluorescent images of astrocytes cultured on MEA at 35 DIV. Immunocytochemistry of GFAP (green), human nuclei (red), cell nuclei by Hoechst 33258 (blue), merged images in (A) and MAP2 (red) in (B). Scale bar = 200 μm. C Representative oscillation waveform at the spontaneous activity measurement for 10 min at 14 DIV. (D) The magnified waveform of the red underlined time in (C). (E) Plot of maximum amplitude in 10 min oscillation waveform of each well. Error bars indicate the SEM. [file Image_1.TIF]
